# The Importance of Country-of-Origin Information on Food Product Packaging

**DOI:** 10.3390/nu13093251

**Published:** 2021-09-18

**Authors:** Paweł Bryła

**Affiliations:** Department of International Marketing and Retailing, Faculty of International and Political Studies, University of Lodz, Narutowicza 59a, 90-131 Lodz, Poland; pawel.bryla@uni.lodz.pl; Tel.: +48-426655830

**Keywords:** country-of-origin (COO), country-of-origin effect (COOE), country-of-origin labeling (COOL), food marketing, food labeling, consumer behavior, consumer ethnocentrism

## Abstract

This study aimed to identify selected predictors of country-of-origin (COO) information placed on food packaging. The dependent variable was operationalized in two ways: (1) as a Likert-style question about COO importance in general, and (2) as an indication of COO as the most important food attribute at first purchase, which I called top-of-mind COO importance. The survey was conducted with the use of the internet panel of a research agency in a representative sample of 1051 Polish consumers. In bivariate analyses, I identified the characteristics of consumer segments attaching high importance to each type of COO information. In a multivariate log-normal regression, general COO importance was affected to the largest extent by the product originating from Poland, which confirmed the strong relation between COO importance and consumer ethnocentrism. In multivariate logit regressions, top-of-mind COO importance depended also on the Polish origin of the product to the largest extent. The remaining predictors were sex (men were over 1.5 times more likely to indicate COO as the most important attribute) and age (each year of life contributing to a 2% increase in the likelihood of indicating top-of-mind COO). A theoretical implication is to differentiate between general and top-of-mind COO measures, as different results were obtained depending on whether the COO effect was measured as a response to questions such as “How important is the product COO for you?” or “What is the most important product attribute for you?—COO” Not only were the answer patterns different, but their determinants also varied.

## 1. Introduction

Country-of-origin (COO) information constitutes one of the principal attributes used by consumers in their process of selecting a food product. Despite a relatively long tradition of research in this subject area, there is still insufficient knowledge about COO food-labeling effects [1]. Even though they are among the most commonly traded items, food products have received less attention from COO researchers than other product categories (i.e., consumer electronics, cars, fashion, and footwear). Furthermore, most of the research that focuses on food was conducted before the introduction of mandatory labeling requirements in many important markets [2]. Consumer assessments of country-of-origin, brand, and price cues are connected [3], but a country’s image may differ across product categories [4]. Indication of origin may become a signal of enhanced quality depending on the association of the source-of-origin with higher food safety or quality [5]. COO seems to become less significant when other quality cues are salient [6]. The effects of COO disclosure were attenuated by the presentation of objective information about the food processing systems of competing countries [7]. Consumers that access COO labels often misinterpret this information [8]. The COO effect is different for ‘made in’ and brand-origin countries [9]. Information technology can increase the convenience of verifying COO information for consumers [10]. The COO effect is related to consumer ethnocentrism. Consumers who tend to distrust other people are more likely to avoid imported products [11]. The intensity of ethnocentric attitudes towards food is differentiated by sociodemographic variables, such as: age, education, and assessment of the financial situation [12]. The importance of country-of-origin in product evaluation correlates positively with consumer ethnocentrism or animosity only among frequent purchasers [13]. National and regional ethnocentric attitudes should be differentiated [14,15]. Many producers try to stress the national and regional character of their products (mainly food) because it is an effective incentive for purchasing decisions [16].

In spite of a large and growing body of research on the COO effect, there is a lack of studies on this phenomenon on the food market in Poland, based on nationally representative samples. This study aimed to address this research gap and answer the following research question: what are the characteristics of consumers who attach importance to country-of-origin information placed on food labels? A wide range of demographic, behavioral, and psychographic criteria were tested, first in analyses of variance, and second, in regression models in order to find out which predictors of the dependent variable were the most important. Two types of country-of-origin information were examined: the general declared importance of this information, and selecting this criterion as the most important attribute at the first purchase of a food product.

## 2. Materials and Methods

The study was conducted with the use of the CAWI (Computer Assisted Web Interviews) method in 2018. A specialized research agency was commissioned by the University of Lodz to administer the survey. I designed the questionnaire on the basis of scales validated in previous research [17,18,19,20,21,22,23,24,25,26,27,28,29]. The respondents were informed about the solely scientific purpose of the research and the respect of the principle of anonymity.

The research setting was Poland, a large Central European country which was rapidly developing within the European Union before the coronavirus pandemic. Polish consumers were sensitized to the need to support local producers by numerous social campaigns. Many retailers emphasized the Polish origin of food products in their marketing communications.

The sample size was 1051 persons. Quota sampling was applied regarding the following criteria: sex (males and females), age (the following age intervals: 15–24, 25–34, 35–44, 45–54, 55–64, and 65 and more), education (primary, secondary, tertiary), place of living (urban and rural areas), and voivodship (all 16 Polish regions). Thanks to this approach, the structure of the sample resembled the general population of Polish consumers according to the aforementioned criteria. The sample characteristics according to these criteria were presented in Table A1 in a previous publication [30].

I provide some further information about the sample characteristics which may be useful for researchers undertaking similar studies in other countries. The structure of the sample by the number of household members was as follows: 1—9.5%, 2—31.7%, 3—24.6%, 4—19.1%, 5—7.7%, 6 and more—7.3%. The structure of the sample according to the number of children in the household was as follows: 0—52.4%, 1—25.1%, 2—16.9%, 3—3.8%, 4—1.2%, 5 and more—0.5%. The structure of the respondents according to their professional activity was as follows: white-collar worker—13.3%, blue-collar worker—28.0%, unemployed—4.7%, student—10.5%, not working and caring for the family—9.5%, old age pensioner or disability pensioner—29.7%. The structure of the sample according to monthly disposable income net of tax of the whole household was as follows: below PLN 2000—15.0%, PLN 2001–3000—23.8%, PLN 3001–4000—21.9%, PLN 4001–5000—18.2%, PLN 5001–6000—10.5%, over PLN 6000—10.7%.

The dependent variable: importance of country-of-origin information on the food product packaging was operationalized in two ways. First, it was measured as an answer to the following question: “How important for you is the following information on the food product packaging? Country of origin”, with the following answer options: very important, rather important, average, rather not important, with no importance, which were coded in the 5–1 scale. I will refer to this measure as COO importance. Second, it was measured as an answer to the following single-choice question: “What is the most important type of information on the label (with the exception of price) when you buy a food product for the first time?”, with seven answer options: (a) country of origin, (b) nutritional information, (c) information about health effects, (d) list of ingredients, (e) expiry date, (f) other, (g) I don’t know. I will refer to this measure as top-of-mind COO importance.

In order to analyze the collected empirical material, t-tests, analyses of variance (ANOVAs), Spearman correlation coefficients, multivariate log-normal regression models, and bivariate and multivariate logit regression models were applied. I used Statistica 12.0 (TIBCO Software Inc., Palo Alto, CA, USA) software to conduct the statistical analyses.

Ethical review and approval were waived for this study, because according to the Work regulations of the Committee for Research Bioethics of the University of Lodz (attachment to Ordinance No. 149 of the Rector of the University of Lodz of 15 July 2013), the consent of this committee was not required for the kind of studies conducted in this research project. The consent is necessary for biological, medical, chemical, and physical research projects using biological material collected from humans, as well as research interfering with the human psyche. My survey did not fall into these categories.

## 3. Results

18.8% of respondents indicated the country of origin as a very important type of information on the food packaging, whereas 32.2% considered it rather important, 35.4% of average importance, 10.5% rather unimportant, and 3.1% with no importance. Therefore, for the majority of respondents, this attribute was important, and the segment of those who attach very high importance to it was considerable (almost 1/5 of all respondents). In the 1–5 scale, the importance of country-of-origin information amounted to 3.531 on average, taking the 7th rank out of 11 types of information placed on labels which were subject to respondents’ evaluation. The most important types of information were: expiry date, list of ingredients, and price.

Women attached significantly higher importance to COO information on the food packaging than men (3.600 vs. 3.452, t = 2.368, *p* = 0.018). Age also significantly differentiated COO importance (F = 10.391, *p* < 0.001), with the highest level of this measure observed in the age group of 55–64 years old (3.763) and the lowest among the youngest consumers (3.032). The region had no significant impact on the dependent variable (F = 1.159, *p* = 0.298). The place of living, understood as the size of the city, did not influence COO either (F = 1.092, *p* = 0.352). Education level played an important role, with those having primary education evaluating COO importance significantly lower than better educated buyers (F = 6.645, *p* < 0.001). Professional activity was also found to differentiate COO importance (F = 8.332, *p* < 0.001), with the highest levels observed among inactive respondents—not working and taking care of one’s family (3.670), and old age or disability pensioners (3.644), and the lowest among students (2.982), which may be related to age. Family income did not influence COO importance (F = 1.233, *p* = 0.292). Although the household size did not have a significant impact on COO importance (F = 1.233, *p* = 0.292), the number of children in the household did (F = 3.461, *p* = 0.008). The lowest importance of COO was reported in families with four or more children. COO importance was significantly higher among those who purchased organic food than those who did not (t = 7.427, *p* < 0.001) and among buyers of functional food compared to non-buyers (t = 3.003, *p* = 0.003). Buying dietary supplements (t = 1.694, *p* = 0.091) and fair-trade products (t = 1.031, *p* = 0.303) did not have a significant impact on COO importance. Neither Body Mass Index (BMI) (F = 1.564, *p* = 0.196), nor self-rated health (F = 0.589, *p* = 0.555) differentiated COO importance significantly. However, self-rated healthiness of one’s diet (F = 16.460, *p* < 0.001) and one’s knowledge about healthy nutrition (F = 8.919, *p* < 0.001) influenced COO importance. Being on a special diet for health reasons also increased COO importance (t = 1.973, *p* = 0.049).

COO importance turned out to correlate significantly with the importance attached to all other investigated types of label information, with the highest Spearman correlations for the organic certificate (ρ = 0.442, *p* < 0.001), health claims (ρ = 0.389, *p* < 0.001), and quality signs (ρ = 0.386, *p* < 0.001). It was also significantly correlated with various types of information used in marketing communications for food products, especially the product originating from Poland (ρ = 0.495, *p* < 0.001), a traditional method of production (ρ = 0.360, *p* < 0.001), and care for the natural environment (ρ = 0.314, *p* < 0.001). It was not correlated only with low-price communication (ρ = 0.036, *p* > 0.05). Furthermore, COO importance was found to be correlated with various measures of reading food labels, namely, reading back-of-package (BOP) labels in the shop (ρ = 0.139, *p* < 0.001), front-of-package (FOP) labels at home (ρ = 0.092, *p* < 0.01), and BOP at home (ρ = 0.131, *p* < 0.001). However, it was not correlated with reading FOP labels in the shop (ρ = 0.048, *p* > 0.05). Unsurprisingly, COO importance was also strongly related to indicating COO as the most important attribute at the first purchase (top-of-mind COO importance) (t = 7.802, *p* < 0.001). Bivariate analyses allowed the distinction of the characteristics of consumer segments attaching the greatest importance to country-of-origin information on food labels (Table 1). It turned out that such consumers were most likely to be women aged 55–64, living in the rural areas, not working, but having above-average family income, having one child, being overweight, having average self-rated health, assessing one’s dietary knowledge as large, assessing one’s diet as healthy, and buying organic food.

In a multivariate log-normal regression model (Table 2), 31 independent variables were included, nine of which turned out to significantly influence COO importance. These predictors were as follows: information about the product originating from Poland (regression coefficient (β) = 0.115, standard error (SE) = 0.010, Wald χ^2^ = 132.920, *p* < 0.001), communicating the utility of the product in a particular diet (β = −0.047, SE = 0.010, χ^2^ = 24.383, *p* < 0.001), importance of the brand information on the packaging (β = 0.042, SE = 0.009, χ^2^ = 23.737, *p* < 0.001), organic certificate (β = 0.044, SE = 0.010, χ^2^ = 20.336, *p* < 0.001), communicating above-average quality of the product (β = −0.033, SE = 0.012, χ^2^ = 8.143, *p* = 0.004), having vocational education compared to primary education (β = 0.071, SE = 0.038, χ^2^ = 6.259, *p* = 0.012), quality signs on the label (β = 0.023, SE = 0.010, χ^2^ = 5.302, *p* = 0.021), communicating care for the natural environment (β = 0.021, SE = 0.010, χ^2^ = 4.393, *p* = 0.036), and diet healthiness (β = −0.026, SE = 0.013, χ^2^ = 4.098, *p* = 0.043). It is worth noting that in the multivariate model, diet healthiness was negatively related to COO importance, contrary to our previous bivariate analyses. COO importance was also negatively affected by importance attached to marketing communications about the utility of the product in a particular diet. COO importance was determined to the largest extent by the importance of the product originating from Poland, as reflected both in the absolute value of the regression coefficient and Wald χ^2^. This regression model had satisfactory goodness-of-fit indices: Pearson χ^2^(964) = 562.5, AIC = 2352.9, BIC = 2559.2, Log(LR) = −1134.4.

As far as top-of-mind COO importance was concerned, country-of-origin information was indicated as the most important at the first purchase by 12.4% of respondents. It had the third rank, following the expiry date, and list of ingredients.

Top-of-mind COO importance was significantly higher among men than women (14.87% vs. 10.18%, Yates χ^2^ = 4.883, *p* = 0.027), and it was differentiated by age (χ^2^ = 29.581, *p* < 0.001), with the highest level in the 55–64 years-old group (21.82%) and the lowest among those aged 15–24 (3.80%). If we take age as a continuous variable, the mean age of those who selected top-of-mind COO importance was 51.7 years, while the mean age of those who did not was 44.1 years (t = 4.684, *p* < 0.001). Top-of-mind COO importance was not differentiated by region (χ^2^ = 15.985, *p* = 0.383), place of living (χ^2^ = 1.968, *p* = 0.579), education level (χ^2^ = 5.771, *p* = 0.123), or income (χ^2^ = 8.605, *p* = 0.126), but it varied significantly by professional activity (χ^2^ = 12.412, *p* = 0.030), with students displaying the lowest level (2.73%). It also depended on household size (χ^2^ = 11.155, *p* = 0.048), with the highest level in single-person households, and the lowest in families composed of 4 members. The number of children did not differentiate it significantly (χ^2^ = 3.613, *p* = 0.461). It was not associated with purchasing habits regarding dietary supplements (χ^2^ = 0.010, *p* = 0.919), organic food (χ^2^ = 1.378, *p* = 0.241), functional food (χ^2^ = 0.267, *p* = 0.606), or fair-trade products (χ^2^ = 1.049, *p* = 0.306). Top-of-mind COO importance increased systematically with the BMI intervals (underweight, normal, overweight, and obese) (χ^2^ = 8.182, *p* = 0.042), but was not differentiated by the self-rated health (χ^2^ = 0.452, *p* = 0.798), diet healthiness evaluation (χ^2^ = 1.727, *p* = 0.422), self-rated diet knowledge (χ^2^ = 1.550, *p* = 0.461), and being on a special diet for health reasons (Yates χ^2^ = 1.490, *p* = 0.222). Our bivariate analyses enabled the distinction of the characteristics of consumers attaching the greatest importance to COO information at the first purchase of a food product (Table 3). These characteristics differed from those of consumers declaring a high importance of COO information on the food label in general. Here, top-of-mind COO importance was the highest among men living in small towns, being old age or disability pensioners, living alone, being obese, with poor self-rated health, small dietary knowledge, and average diet healthiness.

Since top-of-mind COO importance was conceptualized as a dichotomous variable in this study, logit regressions were appropriate to investigate its predictors. In bivariate logit regressions (Table 4), nine variables turned out significant: sex (being a man increased the probability of selecting this option—odds ratio OR = 1.541, *p* = 0.022), age (OR = 1.026, *p* < 0.001), professional activity (being a student compared to a blue-collar worker—OR = 0.178, *p* = 0.005), household size (OR = 0.833, *p* = 0.009), BMI (OR = 1.042, *p* = 0.015), importance attached to care for the natural environment (OR = 1.422, *p* = 0.001), importance attached to supporting producers (OR = 1.413, *p* = 0.001), importance attached to the product originating from Poland (OR = 2.433, *p* < 0.001), and importance attached to a traditional method of production (OR = 1.740, *p* < 0.001).

In a multivariate logit regression model (Table 5), only three predictors remained significant: sex, with being a woman as a reference (OR = 1.681, *p* = 0.023), age (OR = 1.028, *p* = 0.008), and the importance attached to the product originating from Poland (OR = 2.229, *p* < 0.001). The Hosmer–Lemeshow test for this model was satisfactory at χ^2^ = 12.396, *p* = 0.134.

In order to arrive at a more parsimonious model explaining the dependent variable, I opted for running a retrograde stepwise logit regression (Table 6). Here, the same 3 predictors remained significant as in the previous model, but the model had more favorable Hosmer–Lemeshow statistics: χ^2^ = 7.130, *p* = 0.523. Attaching a higher importance to the Polish origin of food products led to indicating COO information as the most important message at the first purchase. Being a man increased top-of-mind COO importance by over 50%, and each year of one’s life increased it by 2% on average.

## 4. Discussion

I identified selected predictors of the importance attached to country-of-origin information on food packaging. Two measures of this importance were distinguished: COO importance and top-of-mind COO importance. COO importance was found to increase significantly when the respondent had vocational education rather than primary education, and to decrease with self-reported diet healthiness. It grew with the importance attached to the following types of information put on food labels: brand, organic certificate, and quality signs. Unsurprisingly, it was determined to the largest extent by the importance attached to the product originating from Poland, which confirmed the strong link of COO importance with consumer ethnocentrism. The preference for domestic food products, especially those originating from the same region where the consumer lives [15], may be considered a pattern of sustainable consumption or sustainable diet. Finally, COO importance decreased with such types of messages in the marketing communication as: the utility of the product in a particular diet, and above-average quality of the product. It is worth noting that COO importance depended mainly on psychographic rather than demographic criteria, especially the attitude to certain other types of information on the product label and in the marketing communication. Nevertheless, top-of-mind COO importance turned out to depend only on three variables, two of which were demographic: sex and age, and the third being an ethnocentric attitude. This is consistent with previous research that demonstrated that ethnocentrism affects the perceived quality of domestic and foreign products, leading to the appearance of the COO effect [31]. A Polish study showed that the preferred COO of a brand was the home country [32]. The capability of consumer ethnocentrism in explaining consumer bias in favor of domestic products depends both on the COO and the product category [33]. COO matters for low-involvement products, but other extrinsic cues (price and brand) may prevail over the COO effect [4]. In the case of some developing countries such as China, there is an opposite effect—consumers prefer imported food, because it is perceived to be of higher quality than domestic food [34]. Another study revealed a general preference for domestic over imported organic food products, with exceptions to the latter in emerging markets [35].

The main contribution of this paper stems from identifying different predictors of the COO effect on the food market from two perspectives: general evaluation and top-of-mind attribute selection, in a large-scale representative sample of Polish consumers. The theoretical implications of this study include the suggestion to differentiate between general and top-of-mind COO effect measures, and to analyze this phenomenon in the broader context of accompanying information put on labels and marketing communication messages related to a given product. It is worth noting that being a man significantly increased top-of-mind COO, whereas women tended to indicate higher general COO importance. This difference was statistically significant (t = 2.368, *p* = 0.018), as demonstrated in my previous research [36]. The managerial implications are to associate COO communication with branding, presenting organic certificates and quality signs on the label, and emphasizing an ecological attitude of the enterprise in its marketing communication.

This study in not devoid of limitations which open avenues for future research. One possibility could be to differentiate between various kinds of COO, especially country of processing, country of raw materials, country of brand origin, and country of the company ownership. Second, more disaggregated approaches to different product categories on the food market are possible. Third, the research setting was a single country: Poland. Although it is a European Union member, which entails the similarity of the legal environment with other member states, cultural, political, and socio-economic differences may come into play in international comparisons.

## Figures and Tables

**Table 1:** Consumer segments attaching the highest importance to country-of-origin information.

Criteria	Segments
Sex	Women
Age	55–64
Region	Świętokrzyskie
Place of living	Rural areas
Education	Vocational
Professional activity	Not working and taking care of one’s family
Income	PLN 4001–5000 (per month, for the whole household)
Household size	3
Number of children	1
Buying supplements	Yes
Buying organic food	Yes
Buying functional food	Yes
Buying fair-trade products	Yes
Body Mass Index	Overweight
Self-rated health	Average
Diet healthiness	Healthy
Diet knowledge	Large
Being on a special diet	Yes

**Table 2:** Selected predictors of the importance of country-of-origin information on the food packaging (a multivariate log-normal regression model).

Independent Variables	Coeff.	SE	Wald χ^2^	*p*
Age	0.001	0.001	0.866	0.352
Diet healthiness	−0.026	0.013	**4.098**	**0.043**
Diet knowledge	0.005	0.012	0.178	0.673
Health claims	0.013	0.011	1.302	0.254
Nutrition claims	0.021	0.011	3.486	0.062
List of ingredients	0.013	0.010	1.868	0.172
Expiry date	0.020	0.011	3.669	0.055
Cooking recipes	0.015	0.008	3.757	0.053
Brand	0.042	0.009	**23.737**	**<0.001**
Organic certificate	0.044	0.010	**20.336**	**<0.001**
Quality signs	0.023	0.010	**5.304**	**0.021**
Recommendations of scientific institutes	0.013	0.009	2.362	0.124
Price	0.002	0.008	0.035	0.851
Health effects of eating a given product	0.000	0.011	0.002	0.966
Care for the natural environment	0.021	0.010	**4.393**	**0.036**
Supporting producers (e.g., farmers)	−0.011	0.010	1.392	0.238
The product originating from Poland	0.115	0.010	**132.920**	**<0.001**
The utility of the product in a particular diet	−0.047	0.010	**24.383**	**<0.001**
Above-average quality of the product	−0.033	0.012	**8.143**	**0.004**
Traditional method of production	0.016	0.011	2.034	0.154
FOP in the shop	0.000	0.000	0.059	0.808
BOP in the shop	0.000	0.000	1.353	0.245
FOP at home	0.000	0.000	0.547	0.460
BOP at home	0.000	0.000	0.000	0.990
Sex: woman-reference	−0.007	0.015	0.231	0.631
Education: primary-reference				
Vocational education	0.071	0.028	**6.259**	**0.012**
Secondary education	0.034	0.028	1.435	0.231
Tertiary education	0.037	0.032	1.360	0.244
Professional activity: blue-collar-reference				
White-collar worker	0.019	0.025	0.558	0.455
Unemployed	−0.002	0.034	0.004	0.951
Student	−0.033	0.033	0.973	0.324
Not working and taking care of one’s family	0.031	0.026	1.402	0.236
Old age pensioner or disability pensioner	0.007	0.023	0.090	0.764
Number of children: 0-reference				
1 child in the household	0.023	0.017	1.888	0.169
2 children in the household	−0.021	0.021	0.995	0.319
3 children in the household	0.016	0.039	0.161	0.688
4+ children in the household	−0.094	0.064	2.190	0.139
Purchasing organic food	0.019	0.015	1.467	0.226
Purchasing functional food	−0.001	0.015	0.004	0.947
Being on a special diet for health reasons	0.034	0.018	3.495	0.062

SE: standard error. The bold is used to statistically significant results (*p* < 0.05).

**Table 3:** Consumer segments attaching the highest importance to country-of-origin information at first purchase.

Criteria	Segments
Sex	men
Age	55–64
Region	Lubuskie
Place of living	Town up to 50,000
Education	Vocational
Professional activity	Old age pensioner or disability pensioner
Income	PLN 4001–5000
Household size	1
Number of children	4+
Buying supplements	Yes
Buying organic food	Yes
Buying functional food	No
Buying fair-trade products	Yes
Body Mass Index	Obese
Self-rated health	Poor
Diet healthiness	Average
Diet knowledge	Small
Being on a special diet	Yes

**Table 4:** Selected predictors of country-of-origin information being the most important type of information on the food label at the first purchase of a product (bivariate logit regressions).

Independent Variables	OR	−95% CL	+95% CL	*p*	LR1 Test	χ^2^	*p*
Sex: woman-reference	1.541	1.065	2.231	**0.022**	**−390.653**	**5.297**	**0.021**
Age (years)	1.026	1.015	1.037	**<0.001**	**−382.331**	**21.941**	**<0.001**
Professional activity: blue-collar worker-reference					**−365.448**	**16.134**	**0.006**
White-collar worker	0.878	0.478	1.610	0.673			
Unemployed	0.564	0.193	1.655	0.297			
Student	0.178	0.054	0.588	**0.005**			
Not working and taking care of one’s family	0.949	0.485	1.857	0.878			
Old age pensioner or disability pensioner	1.098	0.695	1.735	0.688			
Household size (persons)	0.833	0.725	0.956	**0.009**	**−389.628**	**7.347**	**0.007**
BMI (kg/m^2^)	1.042	1.008	1.077	**0.015**	**−390.460**	**5.684**	**0.017**
Care for the natural environment (1–5)	1.422	1.151	1.756	**0.001**	**−387.562**	**11.478**	**0.001**
Supporting producers (e.g., farmers) (1–5)	1.413	1.156	1.728	**0.001**	**−387.389**	**11.826**	**0.001**
The product originating from Poland (1–5)	2.433	1.874	3.159	**<0.001**	**−364.542**	**57.520**	**<0.001**
Traditional method of production (1–5)	1.740	1.389	2.180	**<0.001**	**−380.129**	**26.345**	**0.000**

OR: odds ratio. The bold is used to statistically significant results (*p* < 0.05).

**Table 5:** Selected predictors of country-of-origin information being the most important type of information on the food label at the first purchase of a product (a multivariate logit regression).

Independent Variables	OR	−95% CL	+95% CL	*p*	Wald χ^2^	*p*
Sex: woman-reference	1.681	1.073	2.632	**0.023**	**5.146**	**0.023**
Age (years)	1.028	1.007	1.049	**0.008**	**7.139**	**0.008**
Professional activity: blue-collar worker-reference					6.584	0.253
White-collar worker	0.946	0.495	1.807	0.866		
Unemployed	0.757	0.243	2.361	0.631		
Student	0.629	0.168	2.352	0.491		
Not working and taking care of one’s family	1.463	0.682	3.139	0.329		
Old age pensioner or disability pensioner	0.571	0.309	1.053	0.073		
Household size (persons)	0.965	0.822	1.132	0.660	0.193	0.660
BMI (kg/m^2^)	1.013	0.970	1.058	0.548	0.361	0.548
Care for the natural environment (1–5)	1.136	0.858	1.504	0.372	0.796	0.372
Supporting producers (e.g., farmers) (1–5)	1.000	0.763	1.310	0.999	0.000	0.999
The product originating from Poland (1–5)	2.229	1.597	3.113	**<0.001**	**22.148**	**<0.001**
Traditional method of production (1–5)	1.046	0.772	1.418	0.771	0.085	0.771

The bold is used to statistically significant results (*p* < 0.05).

**Table 6:** Selected predictors of country-of-origin information being the most important type of information on the food label at the first purchase of a product (a retrograde stepwise logit regression).

Independent Variables	OR	−95% CL	+95% CL	*p*	Wald χ^2^	*p*
Sex: woman-reference	1.574	1.051	2.358	**0.028**	**9.790**	**0.002**
Age (years)	1.020	1.007	1.032	**0.002**	**4.841**	**0.028**
The product originating from Poland (1–5)	2.468	1.871	3.256	**<0.001**	**40.906**	**<0.001**

The bold is used to statistically significant results (*p* < 0.05).

## Data Availability

The data presented in this study are available on request from the author.
